# Berberine Nanosuspension Enhances Hypoglycemic Efficacy on Streptozotocin Induced Diabetic C57BL/6 Mice

**DOI:** 10.1155/2015/239749

**Published:** 2015-03-17

**Authors:** Zhiping Wang, Junbiao Wu, Qun Zhou, Yifei Wang, Tongsheng Chen

**Affiliations:** ^1^School of Pharmacy, Guangdong Pharmaceutical University, Guangzhou 510006, China; ^2^School of Chinese Materia Medica, Guangzhou University of Chinese Medicine, Guangzhou 510006, China; ^3^Hubei Key Laboratory of Natural Medicinal Chemistry and Resource Evaluation, Tongji School of Pharmacy, Huazhong University of Science and Technology, Wuhan 430030, China; ^4^Institute of Biological Medicine, Jinan University, Guangzhou 510632, China; ^5^MOE Key Laboratory of Laser Life Science & Institute of Laser Life Science, South China Normal University, Guangzhou 510631, China

## Abstract

Berberine (Ber), an isoquinoline derivative alkaloid and active ingredient of Coptis, has been demonstrated to possess antidiabetic activities. However its low oral bioavailability restricts its clinical application. In this report, Ber nanosuspension (Ber-NS) composed of Ber and D-*α*-tocopheryl polyethylene glycol 1000 succinate (TPGS) was prepared by high pressure homogenization technique. Antidiabetic effects of Ber-NS relative to efficacy of bulk Ber were evaluated in streptozotocin (STZ) induced diabetic C57BL/6 mice. The particle size and zeta potential of Ber-NS were 73.1 ± 3.7 nm and 6.99 ± 0.17 mV, respectively. Ber-NS (50 mg/kg) treatment via oral gavage for 8 weeks resulted in a superior hypoglycemic and total cholesterol (TC) and body weight reduction effects compared to an equivalent dose of bulk Ber and metformin (Met, 300 mg/kg). These data indicate that a low dosage Ber-NS decreases blood glucose and improves lipid metabolism in type 2 diabetic C57BL/6 mice. These results suggest that the delivery of Ber as a nanosuspension is a promising approach for treating type 2 diabetes.

## 1. Introduction

Type 2 diabetes mellitus (DM) is a metabolic disorder characterized by hyperglycemia resulting from relative insulin deficiency and insulin resistance in various tissues. The worldwide prevalence of diabetes is estimated to rise to more than 200 million people and may subsequently reach 300 million by 2025 [1–3]. Several oral therapeutic agents are the first choice treatments of type 2 DM. The purpose of these oral hypoglycemic agents is to ameliorate the underlying metabolic disorder, associated with inadequate insulin secretion, insulin resistance, and increased hepatic gluconeogenesis. However, they have limited efficacy and occasionally produced severe side effects such as weight gain, hypoglycemia, gastrointestinal disturbances, liver injury, heart failure, and bloating [4–6].

Berberine (Ber), an isoquinoline derivative alkaloid isolated from several Chinese medicines, such as Berberidis Radix (Chinese name: Sankezhen), Phellodendri chinensis cortex (Chinese name: Huangbo), Coptidis Rhizoma (Chinese name: Huanglian), and Mahoniae Caulis (Chinese name: Gonglaomu), is commonly used as a quality control marker [7–9]. In recent decades, much focus has been put on its significant antidiabetic activities [10–12]. Previous studies showed that Ber exhibited hypoglycemic properties and exhibited insulin sensitization in type 2 diabetic rats by activation of AMPK [13, 14]. Moreover, Ber can ameliorate diabetic complications, such as cardiac dysfunction, endothelial dysfunction, and nephropathy on diabetic rats [15, 16]. Recent clinical investigations indicated that Ber was a potential and safe hypoglycemic drug to treat type 2 diabetic patients with dyslipidemia at an oral dose of 0.3–0.5 g three times daily [17]. Despite the promising biological effects, Ber is poorly absorbed, resulting in low bioavailability after oral administration. It has been reported that the oral bioavailability of Ber in rats was 0.68% [18]. The low absorption and bioavailability of Ber is still not fully understood. Recently, several studies have proposed some interpretations; since Ber is a lipophobic compound, it is restrained from passing through the membranes of intestinal cells. Secondly, Ber acts as a substrate of several ATP-binding cassette transporters, such as P-glycoprotein (P-gp) and multidrug resistance-associated protein. To circumvent these pitfalls, several strategies, like absorption enhancer, self-microemulsion, and solid lipid nanoparticles, have been used to increase its bioavailability [14, 18–20].

Nanocrystal suspension, nanosuspension (NS) for short, is a carrier-free nanoparticles system containing only pure drug crystal and minimum surfactant and/or polymer for stabilization [21]. Reduction of particle size by nanocrystal technology to the nanoscale usually leads to a significant increase in drug solubility and dissolution rate with an obvious improvement in drug bioavailability. Liversidge and Cundy reported that [22], in the same dosage, danazol NS with the average particle size of 169 nm could obtain the *C*_max⁡_ as high as 3.01 mg/ml and the bioavailability of 82% in beagle dogs, while the commercially available danazol suspension with the average particle size of 10 mm could only obtain the *C*_max⁡_ of 0.20 mg/ml and the bioavailability of 5%. It could be found that NS significantly enhanced the oral absorption of danazol, a poorly water-soluble drug. A few techniques have been used to prepare drug loaded NS, including nanoprecipitation, pearl-milling, high speed homogenization, sonication, and high-pressure homogenization (HPH) [23]. Among these techniques, the HPH method with a high productivity and a lower level contamination which is favorable for implementation of industrial products has shown great superiority over other methods. In this study, we evaluate hypoglycemic activity of Ber-NS relative to efficacy of bulk Ber in streptozotocin induced diabetic C57/BL 6 mice.

## 2. Materials and Methods

### 2.1. Preparation of the Ber-NS

HPH technique was applied to prepare Ber-NS. Briefly, D-*α*-tocopheryl polyethylene glycol 1000 succinate (TPGS, BASF, Germany) of 1.0% and hypromellose (HPMC, Dow Chemicals, Dartford, UK) of 0.5% were dissolved in distilled water. Ber (chloride form, purchased from Aladdin industrial corporation, Shanghai, China) powder of 0.5% was dispersed in the aqueous surfactant solution using high speed homogenization 5000 rpm for 10 min (IKA T18 basic ULTRA-TURRAX, Germany). Then the premix was passed through a Lab HPH (APV-2000, Germany); 5 cycles were performed at 500 bar and 15 cycles at 1500 bar.

### 2.2. Characterization of the Ber-NS

The particle size, polydispersity index, and Zeta potential measurements were performed on a Nano-ZS90 (Malvern Instruments Ltd., Malvern, UK) thermostated at 25°C. The sample was diluted 50 times with bidistilled water before the measurements. Each value reported is the average of three measurements. DSC was recorded on a DSC 204 F1 (NETZSCH, Germany). 5–10 mg of samples were sealed in aluminum pans and heated at 10°C/min from 30°C to 300°C and under N_2_ flow (100 ml/min).

### 2.3. Animal Model and Treatment

Female C57BL/6 mice 7-8 weeks old, body weights 15–20 g, were purchased from the medical experimental animal center of Guangdong Province, China. The National Institute of Health guidelines for the care and use of laboratory animals were followed in all animal experimental procedures. The animals, housed individually in plastic cages, were allowed free access to food and water at all time and were maintained on a 12 h light/dark cycle in a controlled temperature (20 to 25°C) and humidity (50 ± 5%) environment for 1 wk before use. The normal chow diet consisted (as a percentage of total kcal) of 12% fat, 60% carbohydrate, and 28% protein and a high-fat diet (HFD) consisted of 41% fat, 41% carbohydrate, and 18% protein. C57BL/6 mice with normal diets and with HFD are generally used as nondiabetic controls and a diabetic model, respectively [24]. After 4 weeks of feeding, the mice were administered fast for 12 h and then received intraperitoneal injection with 120 mg/kg streptozotocin (STZ, Sigma, St. Louis, MO, USA) in citrate solution (0.1 M citric acid and 0.2 M sodium phosphate, pH 4.5). After 3 weeks, diabetic mice (blood glucose concentrations ≥10.5 mmol/L) were administered Ber-NS, bulk Ber (50 mg/kg/d, BN group, Ber group, *n* = 10), and metformin (300 mg/kg/d, Met group, *n* = 10) via oral gavage daily for 8 weeks or treated with vehicle control (Con group, *n* = 10), respectively. Normal mice as control (Nor group, *n* = 10) were administered citrate buffer alone and treated with vehicle control for 8 weeks. Ber and Met were dissolved in saline. All the mice were allowed to continue to feed on their respective diets until the end of the study. Then, biochemical parameters were performed as described below.

### 2.4. Biochemical Assays

Fasting blood samples collected from the orbital cavity were added into precooled tubes containing EDTA (final concentration 4 mmol/L) and centrifuged at 2,500 ×g for 20 min at 4°C. Plasma glucose, cholesterol, and triglyceride levels were measured with commercial kits (Rongsheng-Biotech Company, Shanghai, China).

### 2.5. Statistical Analysis

Results were expressed as mean ± standard deviation (SD). Student's *t*-test was used to compare the mean differences between samples using the statistical software SPSS version 16.0 (SPSS, Chicago). In all cases *P* < 0.05 was considered statistically significant.

## 3. Results

### 3.1. Particle Size Analysis and Zeta Potential

**Figure 1:**
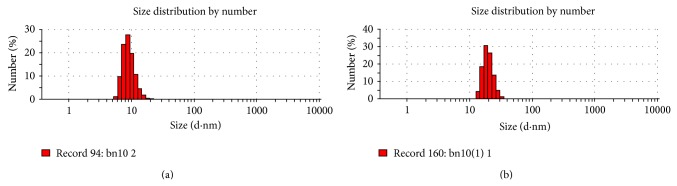
The particles size of Ber-NS ((a) day 0, (b) day 240).

**Figure 2:**
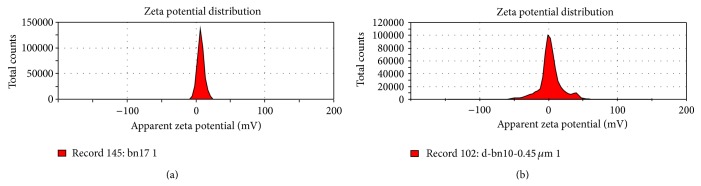
The zeta potential of Ber-NS ((a) day 0, (b) day 240).

The mean particle size and polydispersity index (PDI) were measured immediately after the preparation of the NS. The mean particle size with PDI 0.302 was 72.4 nm. At eight weeks later, the particle size with a PDI of 0.462 was 294.6 nm (Figure 1). The PDI is a measure of particles size distribution. The values less than 0.3 indicate a high degree of homogeneity in particle size and vice versa. The zeta potential of Ber-NS at day 0 and day 240 were 6.95 and 2.5 mV, respectively (Figure 2).

### 3.2. DSC

**Figure 3:**
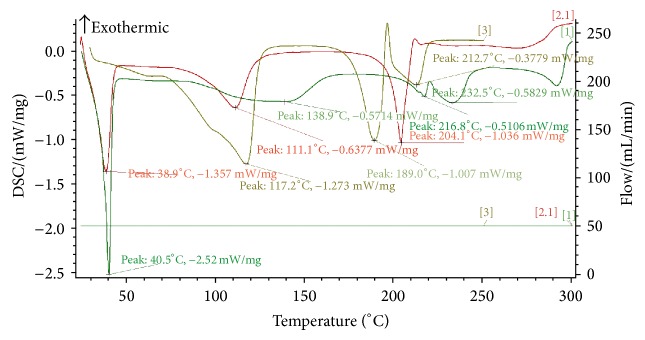
DSC thermograms of [1] Ber-HPMC-TPGS physical mixture, [2] Ber-NS and [3] Ber.

DSC spectrograms are given in Figure 3. The thermogram of Ber showed a sharp endothermic peak at 189°C, corresponding to the melting point of the crystalline form of the drug. In the thermal curve of the physical mixture, the fusion endothermic peak of Ber was much lower than the crystalline substance and shifted to a lower temperature as a consequence of the interaction between Ber, HPMC, and TPGS. However, the thermal curve of the Ber-NS showed a complete disappearance of the Ber endothermic peaks, indicating the formation of an amorphous solid dispersion.

### 3.3. Pharmacodynamics of Ber-NS on Glucose and Lipid Metabolism

To evaluate the effect of Ber-NS on glucose and lipid metabolism, control and diabetic mice were treated with metformin (Met), Ber, Ber-NS, and plasma glucose, total cholesterol, and triglycerides analyzed. As shown in Table 1, diabetic mice exhibited higher fasting blood glucose (FBG), total cholesterol (TC), and triglyceride (TG) levels compared to those of control. Ber (50 mg/kg) treatment decreased FBG levels compared with Con groups. In contrast, BN (50 mg/kg) treatment resulted in a superior hypoglycemic and TC and body weight reduction effects compared to an equivalent dose of bulk Ber and Met (300 mg/kg). These data indicate that a low dosage Ber-NS decreases blood glucose and improves lipid metabolism in type 2 diabetic C57BL/6 mice.

## 4. Discussion

Ber is the major active ingredient of rhizome coptidis, a popular traditional Chinese herb used for the treatment of infection and inflammation. Many animal studies and clinical trials have proved that Ber has significant hypoglycemic effect, even comparable to metformin [14]. Although hypoglycemic effect of Ber is so enticing, it has not yet been used clinically as an antidiabetic drug, mainly because of its low bioavailability (<1%) [18]. As a result, Ber has to be administered repeatedly and at high doses (1500–2000 mg/d) when used in diabetic patients [24]. Although high dose of Ber decreases the blood glucose, it causes major gastrointestinal side-effects, which greatly limits its clinical application. So enhancing the bioavailability of Ber will not only increase its hypoglycemic effect, but also reduce its gastrointestinal side effects. Until recently, there is no multicenter, well controlled, long-term clinical trial to evaluate the efficacy of Ber in the treatment of diabetes, due to its low bioavailability. There were a few reports focusing on the development of new dosage forms of Ber to increase its bioavailability, such as using the intestinal absorption enhancer, self-microemulsion, and solid lipid nanoparticles [14, 18–20]. In this investigation, we first studied the effect of Ber-NS on glucose homeostasis to assess if NS could enhance Ber antidiabetic effects. The present study showed that Ber reduced hyperlipidemia and hyperglycemia in diabetic mice, which were consistent with other studies [13, 14]. FBG was decreased by Ber treatment in type 2 diabetic mice induced by high-fat diet and low dose STZ. The therapeutic effects of Ber-NS were obviously improved when prepared with NS. It showed that the ability of NS to enhance the efficacy of Ber was most probably by improving its bioavailability and increasing its blood level.

More than 95% of the Ber-NS particles showed a small particle size (107.4 nm) (Figure 1). NS were prepared using a HPH method which results in small particles. Small particle size less than 200 nm is desired for being usually invisible to the reticuloendothelial system and for circulating over a prolonged period of time in vivo. Moreover, a zeta potential of ≥±25 mV is recommended for achieving stable dispersions. This is attributed to the existence of repulsive forces between the particles, preventing them from contacting each other and agglomerating. Neutral particles obtained presently can thus be considered intrinsically stable as no interparticulate molecular interactions (both attractive and repulsive) are expected from these particles. In contrast to the neutral and negatively charged particles, the positively charged nanoparticles are taken up more rapidly by the cell membranes [20].

## 5. Conclusion 

In the present study, the low solubility and poor membrane permeability of Ber [25] were enhanced by NS technology. This study demonstrated that Ber-NS possessed excellent antidiabetic activity in diabetic mice models. Moreover, Ber-NS produced a superior hypoglycemic and TC and body weight reduction and less adverse effects compared with bulk Ber and Met. Therefore, Ber-NS may be explored as a novel potential antidiabetic agent for the functional food and pharmaceutical purpose. This study also provides evidences to support the therapeutic effects of compound NS for treatment of diabetes in China. Despite of the promising results from our current investigation, there are still a plethora of practical issues which may be difficult to reconcile for the ultimate use of Ber-NS for the novel target-therapy in diabetes management.

## Figures and Tables

**Table 1:** Changes induced by Ber-NS in biochemical parameter on diabetic C57BL/6 mice.

Group	Dose (mg/kg)	Mice		Body weight (g)		FBG (mmol/L)	TC (mmol/L)	TG (mmol/L)
		Initial	Final	Initial	Final			
Nor	—	10	10	30.1 ± 1.0	31.9 ± 0.7	5.88 ± 0.53	2.39 ± 0.09	0.88 ± 0.10
Con	—	10	10	27.9 ± 1.2	30.7 ± 0.8	8.08 ± 0.85^∗∗∗^	3.34 ± 0.21^∗∗∗^	1.10 ± 0.12^∗∗^
Met	300	10	10	28.6 ± 1.8	33.3 ± 3.6	6.92 ± 0.59^#^	3.13 ± 0.36	0.99 ± 0.16
Ber	50	10	9	28.7 ± 1.6	29.3 ± 3.5	6.81 ± 0.63^#^	2.97 ± 0.70	0.94 ± 0.18
BN	50	10	10	28.6 ± 1.7	32.1 ± 3.4	6.05 ± 0.45^###,Δ^	2.72 ± 0.64^#^	0.97 ± 0.21

Eight weeks after Ber-NS treatment, biochemical parameter of the age-matched normal control group (Nor), STZ induced diabetic group (Con), Metformin treated diabetic group (Met), bulk Ber treated diabetic group (Ber), and Ber-NS treated diabetic group (BN). Data are presented as mean ± SD from 10 animals (*n* = 10) for each group. ^*^ *P* < 0.05, ^**^ *P* < 0.01, ^***^ *P* < 0.001 versus Nor; ^#^ *P* < 0.05, ^##^ *P* < 0.01, ^###^ *P* < 0.001 versus Con; ^Δ^ *P*< 0.05 versus bulk Ber.

## References

[1] King H., Aubert R. E., Herman W. H. (1998). Global burden of diabetes, 1995–2025: prevalence, numerical estimates, and projections. *Diabetes Care*.

[2] Alberti K. G. M. M., Zimmet P. (2013). Epidemiology: Global burden of disease-where does diabetes mellitus fit in?. *Nature Reviews Endocrinology*.

[3] Arredondo A. (2013). Diabetes: a global challenge with high economic burden for public health systems and society. *The American Journal of Public Health*.

[4] Raju B., Resta C., Tibaldi J. T. (2000). Metformin and late gastrointestinal complications. *The American Journal of Medicine*.

[5] Maxion-Bergemann S., Huppertz E., Jacobs L.-D., Müller E., Walleser S. (2005). Improved glycemic control with decreased hypoglycemia prevents long-term complications in type 2 diabetes patients: long-term simulation analysis using the ‘Diabetes Mellitus Model’. *International Journal of Clinical Pharmacology and Therapeutics*.

[6] Patel C., Wyne K. L., McGuire D. K. (2005). Thiazolidinediones, peripheral oedema and congestive heart failure: what is the evidence?. *Diabetes and Vascular Disease Research*.

[7] Dong H., Wang N., Zhao L., Lu F. (2012). Berberine in the treatment of type 2 diabetes mellitus: a systemic review and meta-analysis. *Evidence-Based Complementary and Alternative Medicine*.

[8] Singh I. P., Mahajan S. (2013). Berberine and its derivatives: a patent review (2009–2012). *Expert Opinion on Therapeutic Patents*.

[9] The Pharmacopoeia Commission of the People's Republic of China (2010). *Pharmacopoeia of the People's Republic of China Part I*.

[10] Zhen Z., Chang B., Li M. (2011). Anti-diabetic effects of a coptis chinensis containing new traditional Chinese medicine formula in type 2 diabetic rats. *The American Journal of Chinese Medicine*.

[11] Xia X., Yan J. H., Shen Y. F. (2011). Berberine improves glucose metabolism in diabetic rats by inhibition of hepatic gluconeogenesis. *PLoS ONE*.

[12] Zhang X., Zhao Y., Zhang M. (2012). Structural changes of gut microbiota during berberine-mediated prevention of obesity and insulin resistance in high-fat diet-fed rats. *PLoS ONE*.

[13] Lee Y. S., Kim W. S., Kim K. H. (2006). Berberine, a natural plant product, activates AMP-activated protein kinase with beneficial metabolic effects in diabetic and insulin-resistant states. *Diabetes*.

[14] Zhang M., Lv X. Y., Li J. (2012). Sodium caprate augments the hypoglycemic effect of berberine via AMPK in inhibiting hepatic gluconeogenesis. *Molecular and Cellular Endocrinology*.

[15] Chang W., Zhang M., Li J. (2012). Berberine attenuates ischemia-reperfusion injury via regulation of adenosine-5?-monophosphate kinase activity in both non-ischemic and ischemic areas of the rat heart. *Cardiovascular Drugs and Therapy*.

[16] Tang L. Q., Liu S., Zhang S. T., Zhu L. N., Wang F. L. (2014). Berberine regulates the expression of E-prostanoid receptors in diabetic rats with nephropathy. *Molecular Biology Reports*.

[17] Zhang Y. F., Li X. Y., Zou D. J. (2008). Treatment of type 2 diabetes and dyslipidemia with the natural plant alkaloid berberine. *Journal of Clinical Endocrinology and Metabolism*.

[18] Chen W., Miao Y.-Q., Fan D.-J. (2011). Bioavailability study of berberine and the enhancing effects of TPGS on intestinal absorption in rats. *AAPS PharmSciTech*.

[19] Zhu J. X., Tang D., Feng L. (2013). Development of self-microemulsifying drug delivery system for oral bioavailability enhancement of berberine hydrochloride. *Drug Development and Industrial Pharmacy*.

[20] Xue M., Yang M.-X., Zhang W. (2013). Characterization, pharmacokinetics, and hypoglycemic effect of berberine loaded solid lipid nanoparticles. *International Journal of Nanomedicine*.

[21] Gao L., Liu G., Ma J. (2013). Application of drug nanocrystal technologies on oral drug delivery of poorly soluble drugs. *Pharmaceutical Research*.

[22] Liversidge G. G., Cundy K. C. (1995). Particle size reduction for improvement of oral bioavailability of hydrophobic drugs: I. Absolute oral bioavailability of nanocrystalline danazol in beagle dogs. *International Journal of Pharmaceutics*.

[23] Zhang D., Tan T., Gao L., Zhao W., Wang P. (2007). Preparation of azithromycin nanosuspensions by high pressure homogenization and its physicochemical characteristics studies. *Drug Development and Industrial Pharmacy*.

[24] Yin J., Xing H., Ye J. (2008). Efficacy of berberine in patients with type 2 diabetes mellitus. *Metabolism: Clinical and Experimental*.

[25] Zhaojie M., Ming Z., Shengnan W. (2014). Amorphous solid dispersion of berberine with absorption enhancer demonstrates a remarkable hypoglycemic effect via improving its bioavailability. *International Journal of Pharmaceutics*.

